# Rate of speech affects the comprehension of pronouns in children with developmental language disorder

**DOI:** 10.3389/flang.2024.1394742

**Published:** 2024-08-26

**Authors:** Noelle Abbott, Ignatius Nip, Tracy Love

**Affiliations:** 1 San Diego State University/University of California San Diego Joint Doctoral Program in Language and Communicative Disorders, San Diego, CA, United States; 2 School of Speech, Language, and Hearing Sciences, San Diego State University, San Diego, CA, United States

**Keywords:** developmental language disorder (DLD), child language, rate of speech, pronominal resolution, binding, syntactic dependencies, sentence processing, online and offline processing

## Abstract

This study examined whether children with Developmental Language Disorder (DLD) have knowledge of binding principles (i.e., linking pronouns to their structurally licensed antecedent) during real-time sentence processing (cross-modal priming, real-time) and overt comprehension (sentence-picture matching, interpretative) and whether rate of speech impacted access to that knowledge. Fourteen children with DLD participated in two experiments, with sentences presented auditorily at either a regular or slow speech rate. Sentences were matched except to contain a pronoun, reflexive, or noun phrase (control) in the same syntactic position. Experiment (1) used a cross-modal picture priming paradigm to test real-time pronoun-antecedent linking abilities at both rates of speech. Children were instructed to make a binary decision during the uninterrupted auditory presentation of a sentence to a visually presented image (of the antecedent) at the offset of a pronoun, a reflexive, or a control noun. Response times between conditions (e.g., pronoun vs. control noun) were compared to determine whether participants showed evidence of facilitative priming (faster response times in the pronoun than control noun condition) at either speech rate. Experiment (2) used an auditory sentence-picture-matching task to test final comprehension of similar sentences containing a pronoun or reflexive. Accuracy was compared across both speech rates. For Experiment (1), children with DLD did not show evidence of real-time pronoun-antecedent priming at the regular speech rate. However, when sentences were slowed, they showed facilitative priming for the pronoun condition. For experiment (2), children with DLD performed at-chance when interpreting sentences with pronouns regardless of speech rate. While children with DLD have been shown to have difficulty processing sentences containing anaphors (such as pronouns), results suggest that this is not due to loss of intrinsic knowledge of binding principles. By slowing the rate of speech input, we showed that children with DLD do have access to that knowledge and can make the correct link during real-time processing between a pronoun and its structurally licensed antecedent (Experiment 1) but need more time to do so. However, the effect of slowed speech input does not extend to final comprehension (Experiment 2).

## 1 Introduction

Developmental Language Disorder (DLD; previously referred to as Specific Language Impairment or SLI) is a heterogenous neurodevelopmental disorder that can disrupt expressive and/or receptive language abilities but cannot be attributed to hearing loss, intellectual disability, or neurological damage. DLD is estimated to affect around 7–10% of school-age children making it more prevalent than other neurodevelopmental disorders, such as autism spectrum disorder (ASD) (Norbury et al., 2016; Tomblin et al., 1997; Zablotsky et al., 2019). Despite its prevalence, there has been significant debate around the source of the deficit, especially regarding sentence level comprehension. The three most well-represented arguments in the literature are that the deficits are specific to linguistic knowledge (underlying representations), language processing (such as lexical access or phoneme discrimination), or non-linguistic cognitive processes associated with language (such as working memory or processing speed) (Abbott and Love, 2023; Schwartz, 2017). Therefore, investigations into sentence comprehension impairments in DLD would need to isolate measures of language processing, including components of non-linguistic cognitive processes, from underlying linguistic representations to better understand the source of impairment. Previous studies approached this by comparing processing patterns between typically developing (TD) children and children with DLD on several different language constructions (Borovsky et al., 2013; Montgomery, 2000; Schelletter and Leinonen, 2003; Schwartz et al., 2016). Here, we focus on the processing of complex sentences containing pronouns and reflexives (also known as anaphors) that must refer back to previous noun phrases (also known as antecedents) in children with DLD.

Generative rules of binding (Chomsky, 1982) state that syntactic principles dictate how pronouns and reflexives link to prior occurring noun phrases (i.e., antecedents). According to Principle A, demonstrated in sentence 1a below, the reflexive anaphor *“himself”* must only bind to an antecedent within the same local clause (e.g., “Finn”, as illustrated by the correct sub-index *j* and incorrect sub-index i* for “Ollie”), whereas Principle B, demonstrated in sentence 1b below, states that the pronoun “*him*” must bind to an antecedent outside the local clause, in this case either “Ollie” or some other previously mentioned noun phrase (e.g., “Vera”, “the doctor”, as illustrated by the correct sub-indices *i or k* and incorrect sub-index j* for “Finn”).

Principle A (1a) Ollie_i_ thought [that Finn_j_ helped *himself*_i*/j_].

Principle B (1b) Ollie_i_ thought [that Finn_j_ helped *him*_i/j*/k_]^1^.

Linking pronouns and reflexives to their structurally licensed antecedent requires intrinsic knowledge of binding relationships. However, prior research has produced discrepant results regarding the way in which children demonstrate this knowledge. As has been argued in the literature, young children demonstrate different pronoun-antecedent linking abilities depending upon whether the ability is measured using real-time (temporally constrained, often referred to as *online* methods in the literature) or interpretive (temporally unconstrained, often referred to as *offline* methods in the literature), as each measure captures different aspects of processing (Love et al., 2009; Schwartz et al., 2016).

*Temporally unconstrained* measures allow for additional metalinguistic reflection at all levels of sentence processing including syntactic, semantic, and pragmatic levels. Therefore, results from these tasks measure conscious processing after all linguistic processes are complete (Sato and Vanek, 2023). In contrast, *temporally sensitive* measures provide a glimpse into the moment-by-moment mapping of dependency relationships, thus revealing patterns of comprehension that temporally unconstrained measurements are unable to capture (Love et al., 2009; McKee et al., 1993). Love et al. (2009) demonstrated this difference using a cross-modal picture priming paradigm (Swinney and Prather, 1989) (referred to by the authors as an online measure) and a sentence-picture-matching task (referred to by the authors as an offline measure) to investigate intrinsic knowledge of pronominal binding relationships and final comprehension, respectively (Love et al., 2009). The authors found that when evaluated with a cross-modal picture priming task, TD participants as young as 5 years of age displayed adult-like knowledge of binding principles by correctly linking pronouns to antecedents. However, when the same children were tested using a sentence-picture-matching task, the ability to correctly link a pronoun to its antecedent did not emerge until after 8 years of age, which replicates prior reports in the literature of a maturational effect of pronoun comprehension (Baauw and Cuetos, 2003; Chien and Wexler, 1990; McKee et al., 1993). These results suggest a different developmental trajectory for conscious vs. automatic (subconcious) processing of referential relationships, perhaps due to the additional demands of integrating all levels of linguistic input (syntactic, semantic, etc.), holding that information in memory, and making a final, interpretative decision about sentence meaning. Similar maturational patterns for TD children have been demonstrated with other language constructions such as relative clauses in children 4–6 years of age (Love, 2007), reflexives in children 4;1–6;4 years of age (McKee et al., 1993), and verb-phrase ellipses in children 5–12 years of age (Callahan et al., 2012).

Behavioral studies of binding processes in children with DLD are limited and have resulted in disparate conclusions about the underlying source of language impairment, likely owing to differences in methodology. For example, studies using temporally unconstrained methods have revealed that children with DLD show different developmental trajectories for the acquisition of binding principles when compared to TD children, which has been interpreted as evidence for a deficit in linguistic knowledge, and more specifically in underlying representations (Franks and Connell, 1996; Van Der Lely and Stollwerck, 1997). By contrast, Schwartz et al. (2016) tested binding processes in children with DLD using a temporally sensitive method and found that children with DLD are as accurate as TD children in linking pronouns to their structurally correct antecedents, but are slower to do so (Schwartz et al., 2016). Similarly, Girbau (2017) tested comprehension of direct object pronoun sentences in Spanish-speaking children with DLD using a cross-modal priming paradigm and found that they were slower to respond overall than TD children, but unlike Schwartz et al. (2016) findings, they were also less accurate than their TD peers (Girbau, 2017). Both authors concluded that children with DLD have deficits in processing speed, but it remains unclear whether poorer comprehension abilities are due to domain general processing speed deficits or deficits specific to language processing.

Slower processing of referential relationships suggests that slowing down the rate of speech input may improve the ability to link pronouns and reflexives to their structurally licensed antecedent in children with DLD. A slowed rate of speech has been shown to benefit those with language impairments (Baker and Love, 2023; Love et al., 2008; Pashek and Brookshire, 1982; Poeck and Pietron, 1981). However, slowed input has also been shown to disrupt real-time processing in language unimpaired individuals (Love et al., 2008). In the investigation by Love et al. (2009) looking at rate of speech effects in typically developing (TD) children, the authors explored how slowing down the rate of speech impacted the ability to link pronouns (e.g., *him*) to their structurally licensed noun phrase during real-time sentence processing and final comprehension (Love et al., 2009). The experimental sentences in Love et al. (2009) contained two noun phrases, only one of which was the structurally licensed antecedent of a later appearing pronoun. In addition to their main pronoun sentence condition, the study also included a reflexive condition; a linguistic structure of which typically developing children show early mastery (McKee et al., 1993). In Love et al. (2009), experimental sentences were designed as minimal pairs such that the sentences across all three conditions were identical except for the reference seeking element (pronoun, reflexive, or non-referential noun phrase, NP). Given that the purpose of the study was to test pronoun-antecedent linking, the authors did not directly test on-time reflexive-antecedent linking with these sentences (i.e., they only tested priming for the NP that was structurally linked to the pronoun—i.e., the first noun phrase, NP1). This focused design served two purposes, (1) it allowed the authors to control for additional processing demands introduced by adding a referent to the sentence and (2) it allowed the authors to test whether TD children would *violate* Binding Principle A by incorrectly linking a reflexive with an antecedent outside of its local clause. The authors found that in the *real-time* experiment, TD children showed adult-like patterns of correct referential processing at a regular rate of speech, but when the rate of speech was slowed it disrupted their ability to make those co-referential links, similar to patterns found in neurotypical adults for other syntactically dependent relationships (Love et al., 2008). For the slowed rate of speech condition, where children should have linked the pronoun to the structurally licensed antecedent (NP1), children incorrectly linked the reflexive with NP1, indicating that at the slowed rate of speech, binding principles were incorrectly applied. Conversely, in the portion of the study that tested *temporally unconstrained* final comprehension of sentences containing pronouns, TD children demonstrated difficulty with pronoun-antecedent linking at the regular rate of speech, but with slowed speech input, final comprehension improved. For sentences with reflexives, on the other hand, TD children showed evidence of correctly interpreting sentences containing a reflexive at both speech rates. Based on these findings, and as reflected in Table 1, the authors concluded that TD children showed age-appropriate real-time referential linking for pronouns at the regular speech rate, but pronoun-anaphor linking required additional time (through slowed speech input) for successful interpretive comprehension.

Results from Love et al. (2009) provided insight into rate of speech effects for real-time processing vs. final comprehension of sentences containing pronouns in typically developing children. Additional, research has shown that children with DLD struggle for longer periods of time with correctly assigning pronouns to their antecedents (Baauw and Cuetos, 2003; Van Der Lely and Stollwerck, 1997; Schwartz et al., 2016). It may be the case that children with DLD would benefit from a slowed rate of speech as it would allow for additional time to make the necessary binding links; that is if the linguistic knowledge for binding is available to them. To our knowledge, no studies have investigated this rate of speech manipulation with pronoun processing in DLD. If slowing down the rate of speech improves the ability to link pronouns to their structurally licensed antecedents in children with DLD, it will demonstrate intact knowledge of binding principles, suggesting that the deficit is not in linguistic knowledge or underlying linguistic representations as some theories suggest. Therefore, in the current study, we build on the previously published typically developing findings from Love et al. (2009) by employing the same experimental design and materials to explore whether slowing down the rate of speech will improve real-time and interpretative pronoun-antecedent linking abilities in children with DLD.

### 1.1 The current study

The present study aims to test how rate of speech input impacts referential binding for pronouns during sentence processing (in real-time) and sentence comprehension (after all linguistic input has been processed) in children with DLD. In Experiment (1), we used a cross-modal picture priming paradigm (described below) to determine if children with DLD can correctly link pronouns back to their structurally defined antecedents in real-time with both regular and slow speech input. In Experiment (2), we investigated final comprehension responses to the same stimuli using a sentence-picture matching task at both rates of speech. For the cross-modal priming study (Experiment 1), we hypothesized that at a regular rate of speech, children with DLD would demonstrate impaired pronoun-antecedent linking beyond the age at which TD children are successful (i.e., no evidence of facilitative priming in the pronoun condition, further described in Section 2.1 below), but with a slowed rate of speech input, they would show on-time pronoun-antecedent linking (i.e., facilitative priming in the pronoun condition) upon hearing the pronoun. This pattern would indicate that children with DLD have intact linguistic representations but require more time to make the correct syntactic links. For the sentence-picture matching study (Experiment 2), we hypothesized that children with DLD would show impaired (at-chance) accuracy at the regular speech rate, with small gains in accuracy at the slowed speech rate. Like in Love et al. (2009), this study also included an additional reflexive condition in both experiments to help determine if underlying knowledge of binding principles for both reflexives (Principle A) and pronouns (Principle B) is intact in children with DLD. Prior research has shown that pronoun-anaphor linking takes longer to acquire than reflexive-anaphor linking (Love et al., 2009; Schwartz et al., 2016). Therefore, our hypotheses are focused on the pronoun condition, but we note here that any changes in reflexive-anaphor linking due to the rate of speech manipulation would provide insight into binding knowledge in children with DLD.

The purpose of this study is to build on previously published typically developing findings from Love et al. (2009) to better understand the source of language deficits in children with DLD. Thus, throughout this paper we will be referencing the data from Love et al. (2009) as that publication included neurotypical children within the same age range as those studied here and utilized the same materials and design. The current study only includes children diagnosed with DLD. Important to the design of the cross-modal priming study, each experimental sentence had three versions that were identical minus the inclusion of either a pronoun, a reflexive, or an unrelated noun phrase (see Figure 2 in Section 2.1.2 for example sentences). As will be discussed, the measure of interest is reaction time differences between the matched pronoun and control sentences. By maintaining the structure and wording of the sentence triplets, we were able to isolate the linguistic process of interest, pronoun-anaphor linking, while reducing the effects of other cognitive processes, such as attention and memory. In doing so, this study will provide insight into whether children with DLD have intact knowledge of linguistic principles, or if their deficit stems from some other domain-general linguistic or non-linguistic cognitive process. In other words, this study investigates differences in *real-time* sentence processing and *interpretative* sentence comprehension. Table 1 lists our hypotheses for the pronoun condition at both regular and slow rates of speech for both Experiment 1 and Experiment 2, along with the findings from Love et al. (2009) in gray which are only presented as a reference and not intended as a control group for this work.

## 2 Experiment 1: cross-modal picture priming (real-time sentence processing)

This first experiment in this study investigates how children with DLD process binding relations in real-time by employing a cross-modal picture priming paradigm (Swinney and Prather, 1989). Findings for typically developing children were previously published in Love et al. (2009) but have also been summarized in Table 1. As stated above, these neurotypical findings are not meant to represent a control group for the current study but instead are provided for reader convenience.

### 2.1 Materials and methods

#### 2.1.1 Participants

Eighteen monolingual English-speaking children with DLD were recruited for the study. Data from four participants were removed due to not completing all four testing sessions. The remaining 14 participants (*M*_age_ = 7.80 years, *SD*_age_ = 1.58, eight female) met inclusionary criteria for DLD based on clinical consensus, scoring <1.5 standard deviations below the mean on at least two out of the four core language index subtests of the Clinical Evaluation of Language Fundamentals, Version 4 (CELF-IV) (Semel et al., 2003), and scoring >80 on the Test of Nonverbal Intelligence, Version 3 (TONI-III) (Brown et al., 1997). Participant demographics and diagnostic assessment scores are provided in Table 2. Participants also had normal or corrected-to-normal visual acuity and hearing and had no other reported neurodevelopmental disorders (e.g., ASD), genetic disorders (e.g., Down syndrome), or neurological impairments (e.g., head trauma, epilepsy, etc.). Participants were randomly assigned to either the regular rate of speech or the slow rate of speech condition. Following the data screening procedures described in more detail in the Section 2.1.4 below, there were eight children with DLD in the regular rate of speech group (*M*_age_ = 7.42 years, *SD*_age_ = 1.79; range: 6.17–11.67 years; four female) and six children with DLD in the slow rate of speech group (*M*_age_ = 8.32 years, *SD*_age_ = 1.20; range: 6.92–10.17 years; four female). No significant differences in age, CELF-IV score, or TONI-III score were found between groups, and the age range was similar to Love et al. (2009), which used the same methodology to investigate pronoun binding relationships in TD children (see Table 2) (Love et al., 2009).

Participants were recruited from schools in the San Diego Unified School District and Del Mar School District as well as San Diego State University’s (SDSU) Speech-Language Clinic. After the language and cognitive assessments were completed, the children returned for four testing sessions, each separated by at least 3 weeks and lasting ~1 hour (Experiment 1: 3 visits, Experiment 2: 1 visit). This study was approved under both SDSU and the University of California San Diego’s (UCSD) IRB protocols and all participants were compensated for their time.

#### 2.1.2 Study design and stimuli

For experiment 1, a cross-modal picture priming (CMPP) paradigm was used to test real-time sentence processing (Love, 2007; Love et al., 2009; Swinney and Prather, 1989). Materials were taken from a prior study by Love et al. (2009) with neurotypical children and are described in more detail below. Each rate condition (regular or slow) was designed to be within-subjects; in other words, participants provided data for the anaphor (pronoun and reflexive) and control (non-referential noun phrase) conditions across all items, thus serving as their own controls.

**FIGURE 1:**
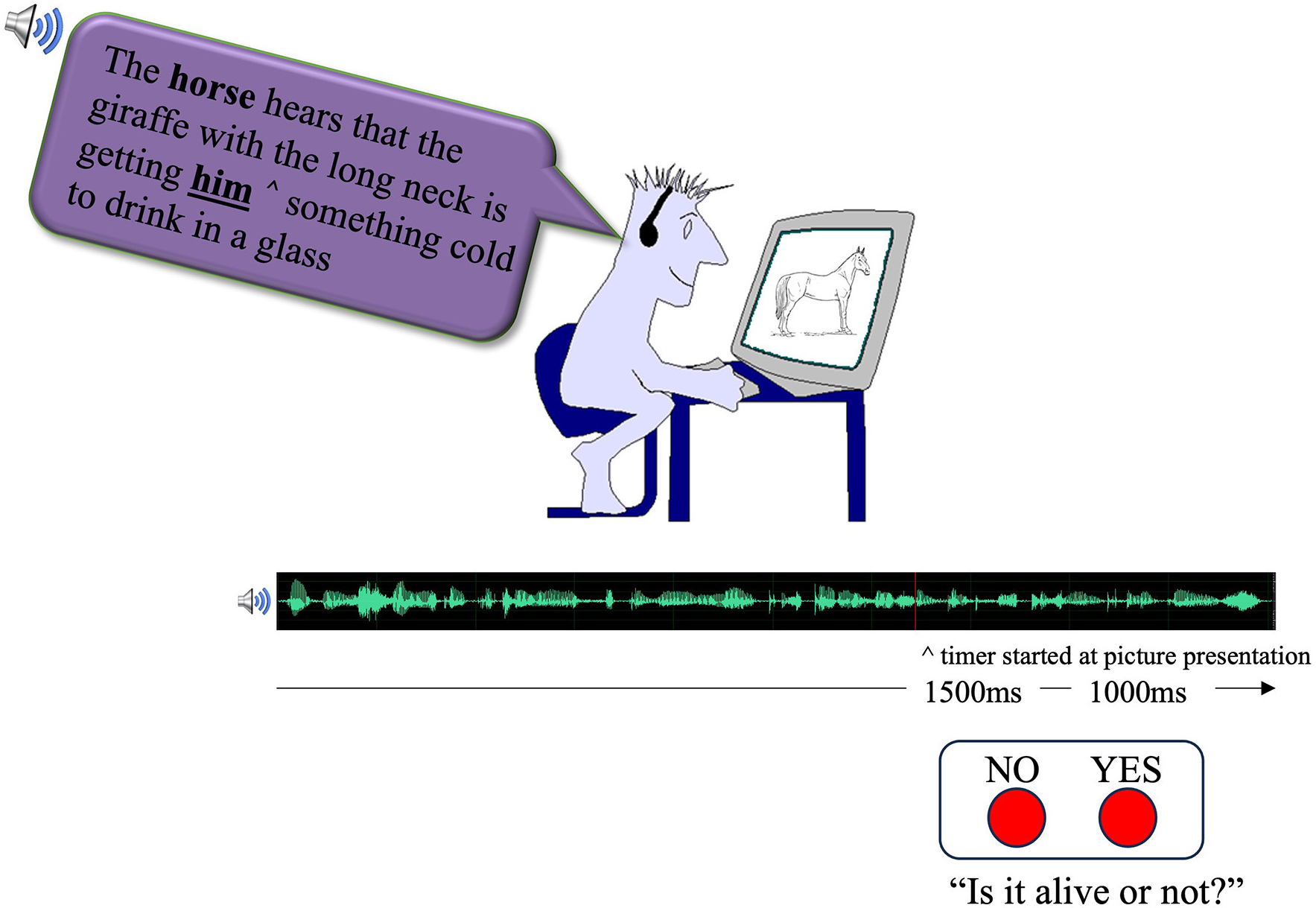
An illustration of the Cross Modal Picture Priming (CMPP) paradigm (Swinney and Prather, 1989). While listening to auditorily presented sentences, participants make a binary (alive or not alive) decision about the picture on the screen. After the presentation of the picture (noted by ^), participants have 1,500 ms to make their decision before the picture disappears from the screen. Following the disappearance of the picture, participants have an additional 1,000 ms to make the decision. Trials were separated by 3,000 ms.

As part of the CMPP paradigm, participants listened to sentences over headphones while seated in front of a computer screen (Figure 1). The children were told that they would be listening to sentences and that at some point during the sentence, a picture would appear, and they would have to use a binary button box to make a decision about whether the picture was “alive” (right button press) or “not alive” (left button press). They were told to try their best to understand the sentences as they would occasionally be asked questions about them. The measure of interest was response time (and accuracy) during the binary decision task. In this CMPP paradigm, the principles of priming dictate that when the image, which is strategically presented at a point of interest in the sentence (e.g., where pronoun-antecedent linking should take place—at the offset of the pronoun), is related in some way to a noun (antecedent) in the sentence, participants should respond faster during the decision task than in a condition where there is no structural link (the control condition), demonstrating evidence of a facilitative priming effect, which reflects the access and processing of the antecedent at that point in time.

**FIGURE 2:**
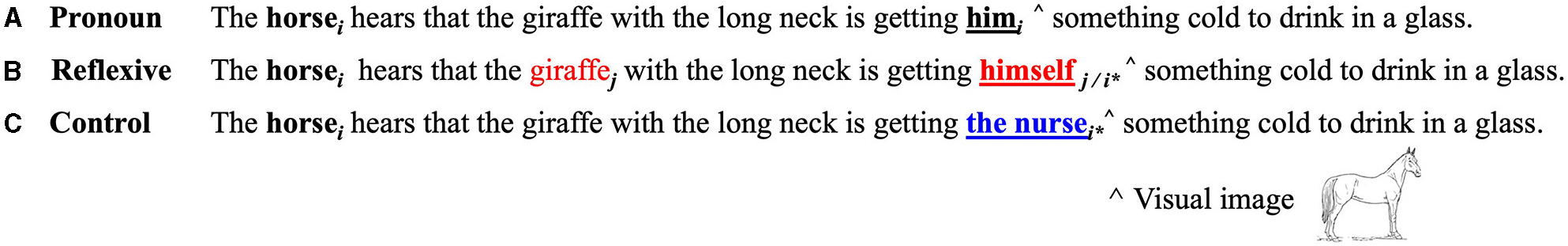
An example of experimental and control items. “The horse” (NP1) is the correct antecedent for only the pronoun condition, noted by the i; i* represents an incorrect link to “The horse”. The “^” represents the point in the sentence that the image of NP1 appears on the screen. *j* indexes the antecedent-anaphor pair for the reflexive condition. Note that participants only saw images of NP1 for the experimental sentences, thus the reflexive is the incorrect anaphor for the visual image.

As described in Love et al. (2009), sentences contained multiple noun phrases. The noun phrases in the experimental sentences were animals, whereas filler sentences contained a mixture of animal and inanimate object names. The sentences for this study included 30 experimental sentences and 30 filler sentences. Each of the 30 experimental sentences were comprised of sentence triplets (10 per condition) that were identical in structure, wording, and length with the exception of the presence of either a pronoun (Principle B), a reflexive (Principle A), or an unrelated non-reference seeking noun phrase (Figure 2). For these experimental items, at the point in the sentence that the child should make a co-referential connection (e.g., pronoun offset) or after hearing the reflexive or unrelated noun, a picture would appear on the computer screen in front of them (noted by the “^” in Figure 2). The children were instructed to respond as quickly and accurately as possible via a binary (alive/not alive) decision when the picture appeared. Importantly, reaction times to the same picture were compared across sentence triplets to gauge priming effects resulting from correct or incorrect linking of the reference seeking elements (pronoun vs. control, reflexive vs. control), to prior occurring noun phrases (NP1). Recall, that linking a reflexive to NP1 would violate Binding Principle A, as reflexives can only bind to antecedents within the local clause. Since NP1 is outside of the reflexive clause, the only appropriate structural link would be to the pronoun. Therefore, children should respond faster to the binary decision task when the image of NP1 appears at the offset of the pronoun rather than the reflexive. Following the pronoun, reflexive, or control noun phrase, there were at least seven syllables to ensure children would have time to process the sentence while simultaneously making a binary decision. All pronoun and reflexive sentences contained third-person singular, masculine pronouns/reflexives, whereas all control sentences contained non-animal female nouns (e.g., the lady).

Given that the images presented for the experimental and control conditions are only of the first noun phrase (NP1, which is structurally linked to the pronoun), our *a priori* hypothesis was that if children with DLD could make accurate binding links in real-time, facilitative priming effects would be present in the pronoun (vs. control) condition only. Said differently, the difference in reaction time between the control and pronoun sentences (control—pronoun reaction time) would be positive (referred to here as positive or facilitative priming). By contrast, positive priming should not be seen in the reflexive condition (2b, in Figure 2, below) as binding for the reflexive should only occur with the second noun phrase (*giraffe*). This mismatch should result in increased reaction times for the reflexive condition when compared to the control condition, possibly due to an inhibition or interference effect. The control condition (2c, in Figure 2, below), with no intra-sentence referential links, was designed to provide a baseline measure of response times for sentences of this type, as children should not be making binding links to prior occurring noun phrases in the sentence. To summarize, quicker response times to the picture of NP1 in the pronoun condition compared to the control condition are interpreted as evidence of positive or facilitative priming, and an indication of correct dependency linking, whereas quicker response times in the reflexive condition (compared to the control condition) are interpreted as evidence of incorrect priming, in other words, a link is made between the reflexive and the wrong NP.

**FIGURE 3:**
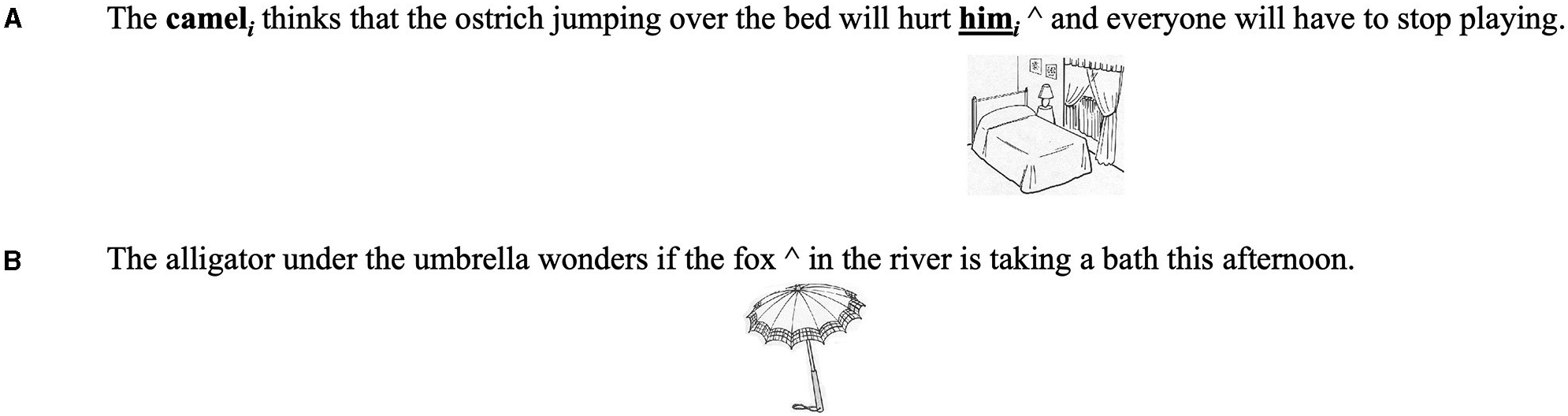
An example of filler items to elicit a “not alive” decision. The “^” represents the point in the sentence that the image appears on the screen.

Filler sentences (*n* = 30) of the same length and complexity as the experimental sentences were included. These sentences were designed to prevent participants from expecting an “alive” response for each picture or using strategies to complete the task. Examples of filler sentences can be seen in Figure 3, below. There were 10 that contained a pronoun or reflexive and 20 that were of another construction.

During each testing session, participants listened to 30 experimental sentences (3 versions per sentence: pronoun, reflexive, non-referential noun phrase) and 30 filler sentences, for a total of 180 items across three visits. Each experimental sentence had a pronoun, a reflexive, and a control version. These were counterbalanced across three scripts, with each script containing only one version of the experimental sentence. For example, referring to Figure 2, (2a) would appear in Script 1, (2b) in Script 2, and (2c) in Script 3 to ensure an equal number of exemplars across scripts and also to prevent any effects from repeating sentences in the same visit. In a given script, no more than 3 experimental sentences were presented in a row. Filler sentences were the same across all three scripts. After participants were trained on the CMPP task (procedure described in more detail below), the experiment began. The first six items presented during each visit were filler items to allow the participant to acclimate to the test, although they were not informed of this. While every participant received all three scripts, one per visit, they were randomly assigned to different script presentation orders.

In the regular rate of speech input condition, sentences were recorded by a native English female speaker and were delivered at an average rate of 4.94 syllables per second. In the slow rate of speech condition, sentences were slowed (while maintaining pitch) to an average of 3.36 syllables per second (using the Cool Edit Pro v1.2 software package; Syntrillium Software, Phoenix, AZ). Participants only heard one rate of speech condition across all three visits. Those in the slow rate of speech condition often reported that the speaker sounded tired.

Stimuli were presented with an in-house software program, TEMPO (version 2.1.2), to control the presentation of the pictures and sentences as well as in the collection of both reaction time and button press response choice. This software has millisecond accuracy and has been used in prior published research with language-impaired and neurotypical child and adult populations (Callahan et al., 2012; Ferrill et al., 2012; Love et al., 2009; Silkes et al., 2020; Sullivan et al., 2017). During the presentation of the sentence, at the predetermined onset of the presentation of the image, a millisecond-accurate computer timer was initiated. The timer terminated when the participant pressed the button to indicate their response on the binary decision task and their button selection and response time was recorded. Each picture remained on the screen for 1,500 ms or until the button press was made (whichever came first). Responses were allowed for an additional 1,000 ms after the picture disappeared, leaving a 2,500 ms window within which a participant could respond. Any response times longer than 2,500 ms were recorded as a no-response and counted as an error. There was a 3,000 ms gap between successive sentences.

#### 2.1.3 Procedure

Following the procedure outlined in Love et al. (2009), participants were trained extensively in order to be able to complete the CMPP task (listening to and comprehending auditorily delivered sentences along with making a button press when shown pictures). Training was delivered in stages that increased in complexity to ensure that participants were not overwhelmed. *First,* the experimenter presented cards with pictures on them, and participants were instructed to verbally respond “yes” or “no” to whether the image was alive (*n* = 7) or not alive (*n* = 7). If the child responded incorrectly, the experimenter would talk through the image until they responded correctly. *In the next stage,* the experimenter used the same set of images and the same task but instructed children to make their “yes” or “no” response using a button box instead of a verbal response. The experimenter continued to provide feedback as needed. *In the next to last training stage*, twenty images were presented on a computer screen in four blocks of five images each. In the first block, images remained on the screen for 1.5 s each and in subsequent blocks they remained on screen for 0.5 s each. This reduced presentation time was meant to encourage participants to respond as quickly as possible, while also maintaining accuracy. Children were instructed to decide whether the image was alive or not alive using a button box and they were trained until they responded correctly to all items. *The final* training protocol introduced children to the cross-modal experimental paradigm in which they simultaneously heard sentences and were told they would see an image during the presentation of the sentence. Their job was to press the correct button as quickly and accurately as possible while listening to the sentence that continued. Participants were trained to 100% response criterion. During this training, experimenters asked comprehension questions to ensure that the children were listening to the sentences. During the first visit, children completed the full training protocol, but during visits two and three, they only completed the final stage to refamiliarize them with the task.

After children completed the final stage of their training, the experimental session began with the six practice filler items followed by presentation of the experimental and remaining filler sentences. As described above, participants were allotted a total of 2,500 ms to make their binary decision. The image remained on the screen for a maximum of 1,500 ms with the image immediately disappearing as soon as the participant made a response during that time. If no response was made by 1,500 ms, the image disappeared, but a decision choice and reaction time could be captured for an additional 1,000 ms. Sentences were separated by 3,000 ms. In addition to this task, the children were also periodically asked comprehension questions about the sentences that they heard to ensure that they were paying attention (18 per session, three attached to filler sentences). Comprehension questions were about nouns heard in the sentence or their location and were not meant to test pronoun-binding relationships. For example, after hearing a sentence like (2a) above, the participant would be asked question (4) below:

(4) Was the sentence you just heard about (A) a horse, (B) an elephant, or (C) a zebra?

Participants indicated their response by either saying their answer out loud or pointing to “A,” “B,” or “C” on an answer sheet.

#### 2.1.4 Analysis

Prior to analysis, four participants (three from the slow rate of speech group and 1 from the regular rate of speech group) were removed from the initial pool of 18 for not completing all three testing sessions. For the remaining 14 participants, all responses to the experimental items that were <250 ms (below the threshold of possible response times) or >2,500 ms (i.e., non-responses; 4% data loss) or were incorrect (wrong button press, 12% data loss) were removed from further analyses. Following this screening, all 14 participants met our threshold of at least 50% of data points remaining. Accuracy on the binary decision task was calculated by taking the number of correct responses out of the total possible responses across all three visits (i.e., 90). A two-way mixed-design ANOVA was conducted using AFEX (Singmann et al., 2023) in R (R Core Team, 2023) to assess differences in accuracy between rate (regular, slow) and within condition (pronoun, reflexive, control).

A univariate screening of reaction times was conducted to identify outliers for each participant per condition. Children with DLD often demonstrate more variable motor responses than TD children (Sack et al., 2022; Vuolo et al., 2017), which means that identifying outliers at the group level could bias the dataset to underrepresent children who are at the high or low ends of RT responses due to motor abilities. Thus, to control for motor variability in our participant’s responses, we mean centered individual response times so that each participant essentially acted as their own control for motor responses. Response times that exceeded 2,000 ms were excluded from further analysis (1.5% data loss) as they do not represent automatic processing. We then calculated the means, 25th, and 75th Tukey hinges for reaction time (RT) by rate and condition for each participant was calculated using PROC Univariate in SAS 9.4 (SAS Institute Inc, 2014). The Tukey hinges were used to identify the upper and lower bounds of the distribution of RT for each participant by rate and condition. Extreme values for each participant were then winsorized to reduce their effects on mean RT values; outliers above the upper bounds were replaced with the values for the upper bounds and a similar procedure for outliers falling below the lower bounds was conducted (Wu and Zuo, 2009). Table 3 shows the proportion of trials that were replaced for each participant.

A mixed linear model with participants and sentences (counterbalanced across all three visits) as random intercepts was conducted to examine the effects of rate (regular, slow), condition (pronoun, reflexive, control), and the interactions of rate × condition on reaction times. In addition, Age was included as a covariate in the model. The model was estimated with SAS 9.4 (SAS Institute Inc, 2014) PROC MIXED. *Post-hoc* testing using least mean squares was used to examine significant main effects and the interaction for the model.

### 2.2 Results

**FIGURE 4:**
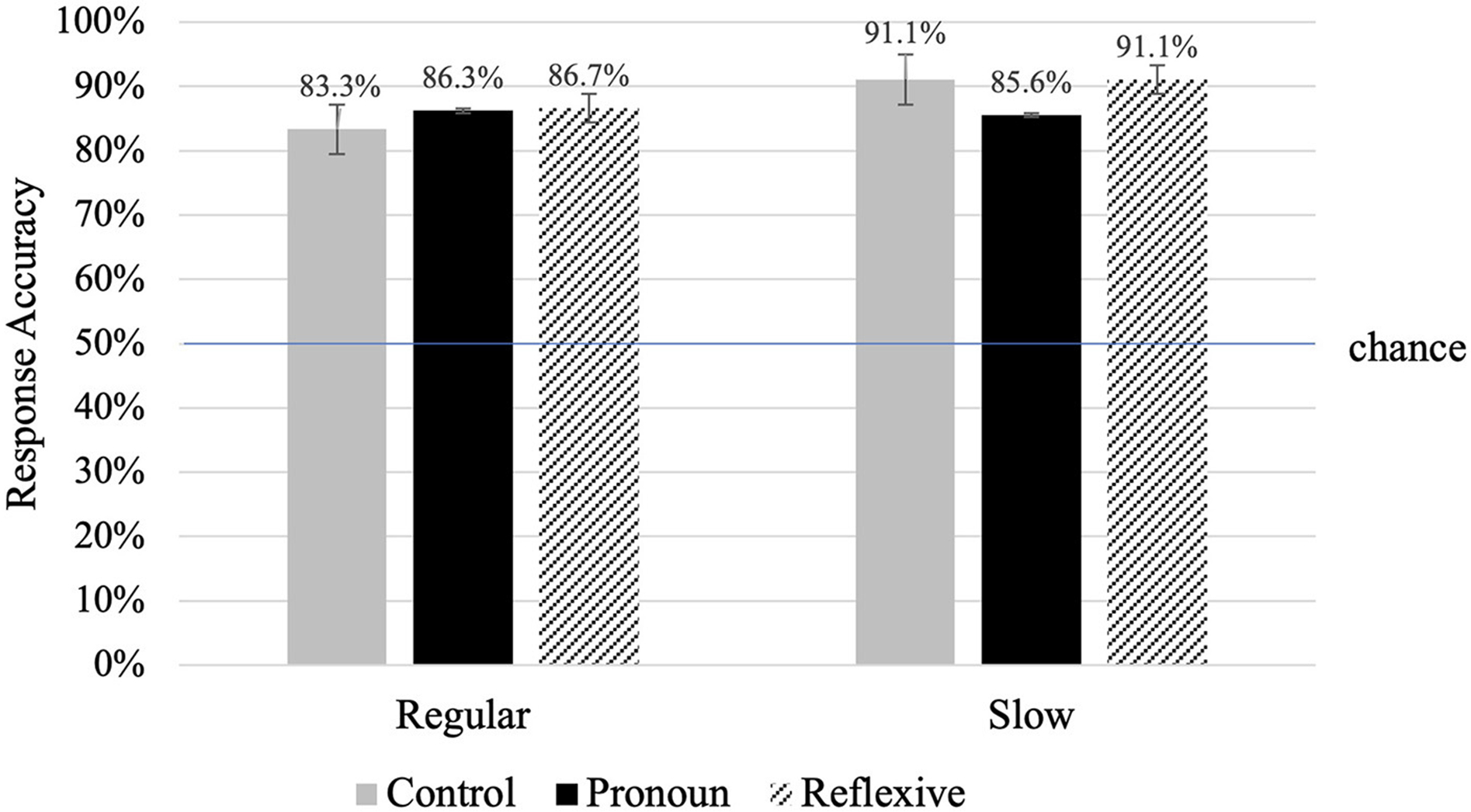
Accuracy (error bars are for standard error) for the binary decision task (alive = yes, not alive = no) following the target prime image. No significant differences between rate, condition, or their interaction were found. Chance is marked by the blue line at 50%.

Accuracy across all experimental conditions and participants for the binary decision task (alive or not alive) was 87.1%. As shown in Figure 4, below, overall accuracy for experimental sentences was well above chance (50%) across all conditions in both the regular [*t*_(7)_ = 6.02, *p* < 0.001] and slow [*t*_(5)_ = 6.41, *p* = 0.001] rate of speech conditions. Importantly, accuracy for filler items was 82%, demonstrating that children were actively making binary decisions throughout the study.

Results from a two-way mixed-effects ANOVA revealed no main effects of rate [*F*_(1,12)_ = 0.28, *p* > 0.05] or condition [*F*_(2,24)_ = 0.88, *p* > 0.05] on accuracy, nor was the rate × condition interaction significant [*F*_(2,24)_ = 1.79, *p* > 0.05], indicating that rate and condition did not significantly impact overall accuracy during this task.

Accuracy was also measured for the three-choice comprehension questions that were scattered throughout the real-time sentence processing portion of the task to ensure participants were not simply ignoring the sentences to respond to the visual targets. For the regular rate of speech input, children with DLD answered the comprehension questions with an average accuracy of 60%, while for the slow condition they answered with an average accuracy of 69.1%. Response averages were above-chance (33%) for both the regular [*t*_(7)_ = 3.86, *p* = 0.006] and slow [*t*_(5)_ = 5.93, *p* = 0.002] rates of speech, suggesting that the children were attending to the auditory input.

Response times for the experimental data can be seen in Table 4. As a reminder, the visual probe in the sentences matched the first main clause noun phrase and appeared at the offset of either the control noun phrase, the pronoun, or the reflexive in the second clause. Therefore, according to binding principles, the picture was the correct antecedent only for the pronoun condition and should facilitate linking the pronoun to the anaphor as indexed by faster response times.

Results from the linear mixed model, revealed a significant main effect of *rate* [*F*_(1,1,090)_ = 12.59, *p* = 0.0004], in which children with DLD responded on average about 173 ms slower overall in the regular speech rate condition (${\bar{x}}_{\text{response time}}=1,044\;\mathrm{ms}$) compared to the slow speech rate condition [${\bar{x}}_{\text{response time}}=871\;\mathrm{ms}$; $t_{\left(1,090\right)}=-3.55$, *p* = 0.0004]. There was also a significant main effect of *age* *F*_(1,1,090)_ = 14.92, *p* = 0.0001) that indicated as children got older, they were quicker to respond to the binary decision task. However, since age was not the primary focus of this study, this pattern, while suggestive, sets the stage for future investigations of rate and age effects on pronoun processing in DLD.

Importantly, the results revealed a significant *rate* × *condition* interaction [*F*_(2,1,090)_ = 8.84, *p* = 0.0002]. To further probe the significant interaction, *post-hoc* testing using least mean squares indicated that children with DLD were significantly faster to respond at the regular rate of speech in the control condition (${\bar{x}}_{\text{response time}}=1,033\;\mathrm{ms}$) compared to (a) the pronoun condition [${\bar{x}}_{\text{response time}}=1,043\;\mathrm{ms}$; *t*_(1,090)_ = −3.64, *p* = 0.0003] and (b) the reflexive condition [${\bar{x}}_{\text{response time}}=1,056\;\mathrm{ms}$; *t*_(1,090)_ = −3.64, *p* = 0.0003], which means there was no evidence of positive priming; that is, the presentation of an image related to the antecedent of the pronoun did not facilitate priming for the pronoun condition (nor did it result in incorrect priming for the reflexive condition) at the normal speech rate.

Prior published literature has indicated that for unimpaired children, the presentation of a stimulus that is related to the noun linked to the pronoun should result in facilitative priming at the offset of the pronoun, whereas an unrelated stimulus should not show a facilitative effect (Clackson et al., 2011; MacDonald and MacWhinney, 1990; Van Der Lely and Stollwerck, 1997). For the pronoun condition in this study, there was no evidence of facilitation for the noun phrase structurally linked to the pronoun (NP1) at the regular speech rate. Instead, results demonstrated that at the regular rate of speech, reaction times were in fact slower for both the pronoun and reflexive conditions compared to the control condition (or negative priming). Given that the noun phrase that was tested should only be linked to the pronoun, the pattern of results for the reflexive condition were expected. However, reaction time patterns for the pronoun condition suggest there was an inhibitory or interference effect. Given that the goal of this study was to evaluate facilitation through semantic priming, the study design was not meant to test or interpret negative priming effects, however, there is always the possibility that the dual task nature of the priming paradigm led to an interference effect. This possibility is unlikely though due to the minimal pair triplet design used in this study. As a reminder, each sentence triplet was identical except for the inclusion of an unrelated noun phrase (control), a pronoun, or a reflexive. At the offset of each condition, participants would see the same image and were asked to make a binary decision about whether the image was of something alive or not alive. Each participant contributed data to all conditions for each of the experimental sentences; that is, they heard all three versions of the sentences across three different visits with the order of sentence presentation randomized across each visit, allowing us to isolate pronoun-antecedent linking while controlling for additional effects of the dual task design. Thus, it is more likely that the negative priming pattern found when comparing reaction time between sentences containing pronouns and those control sentences with a non-referential NP reflects aberrant pronoun-antecedent linking. That is, pronoun-antecedent linking does not occur in real-time with the regular rate of speech input for children with DLD.

**FIGURE 5:**
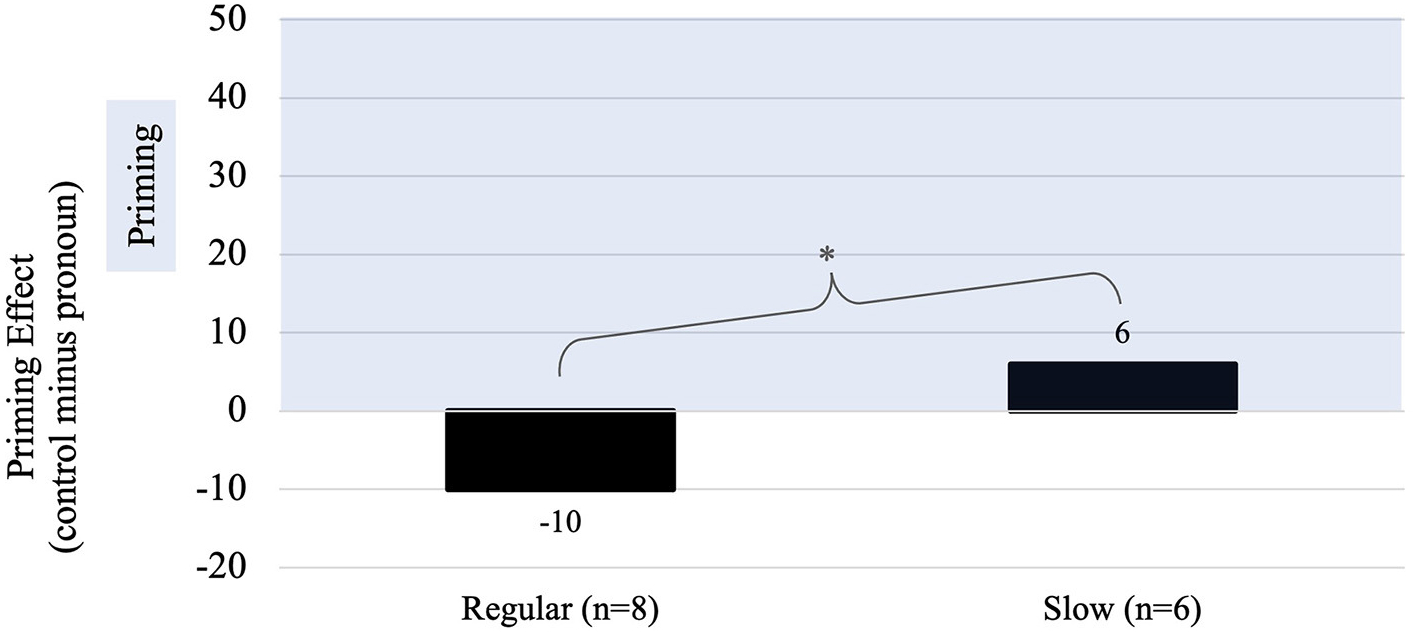
The priming effect (control minus pronoun) for the pronoun condition in both the regular and slow rate speech conditions. A positive difference indicates that the mean RT was faster in the pronoun condition, reflecting facilitative priming (noted by the highlighted region). *The change in RT from the regular to slow rate was significant at *p* < 0.05.

Recall, that our *a priori* hypothesis was that unlike typically developing children, children with DLD would not show evidence of facilitative priming in the pronoun condition at the regular rate of speech (i.e., faster response times in the pronoun condition compared to the control condition) but would do so with slowed speech. To explore our hypothesis, we examined the difference in reaction times to the pronoun and control conditions during the slow speech rate condition as compared to the regular rate condition (Figure 5).

Unlike the priming pattern at the regular rate of speech input described above, *post-hoc* analyses revealed the opposite pattern with the slow rate of speech input. Here, children with DLD were significantly faster to respond in the pronoun condition (${\bar{x}}_{\text{response time}}=879\;\mathrm{ms}$) compared to the control condition [${\bar{x}}_{\text{response time}}=885\;\mathrm{ms}$; $t_{\left(1,090\right)}=2.13$, *p* = 0.03] demonstrating evidence of facilitative priming.

While not the direct focus of this investigation, we also explored whether children with DLD would show a rate effect while processing sentences containing reflexives, which revealed the expected negative priming pattern at the regular rate of speech input. At the slow rate of speech input, while we did not find statistically significant priming, the direction of the reaction time patterns was different from above [slow rate: reflexive ${\bar{x}}_{\text{response time}}=849\;\mathrm{ms}$; control ${\bar{x}}_{\text{response time}}=885\;\mathrm{ms}$; $t_{\left(1,090\right)}=0.19$, *p* = 0.37]. As described in Section 2.3, these patterns of results are consistent with those found with neurotypical children when processing sentences containing reflexives. Specifically, those prior studies showed compliance with Principle A (no incorrect linking) at the regular rate of speech input and disrupted processing at the slowed rate of speech input (Love et al., 2009; McKee et al., 1993).

Overall, results regarding pronoun-antecedent linking from experiment (1) showed no evidence of facilitative priming at the regular speech rate, but when the speech rate was slowed, children with DLD showed evidence of facilitation, suggesting that the linguistic knowledge needed to link pronouns to anaphors (Principle B) during real-time sentence processing is intact, but there is a delay in the speed at which this link is built.

### 2.3 Discussion: experiment 1

Experiment (1) tested response time patterns for linking pronouns and reflexives with the structurally licensed antecedent for the pronoun (the main clause noun phrase, NP1), across two different speech input rate conditions in children with DLD. Here, we were interested in whether children with DLD would show evidence of facilitative or positive priming (indexed by quicker response times to pronouns than controls) when presented with a picture that correctly linked a pronoun with its antecedent; and if rate of speech input modulated this effect. As demonstrated in Love et al. (2009) using the same paradigm and materials, TD children showed evidence of positive pronoun priming (and thus, knowledge of Principle B) during real-time sentence processing at a regular rate of speech (Love et al., 2009). However, when speech was slowed, the positive priming effect went away, indicating that the speech rate impacted the real-time, automatic process of co-referential linking. For this study, we predicted the opposite; that at a regular rate of speech, children with DLD would not demonstrate positive priming effects, but when the speech rate was slowed, their response times would decrease indicating correct linking, as the slowed rate of input would allow for additional processing time to make the link between the pronoun and its antecedent.

Consistent with our predictions, we found that at a regular rate of speech, unlike patterns found with TD children, children with DLD did not show evidence of facilitation (via positive priming), suggesting that they are unable to automatically link pronouns to their antecedents in real-time. However, when the speech input was slowed, children with DLD showed statistically faster response times in the pronoun condition compared to the control condition, indicating facilitative priming. This pattern differs from the results found for TD children in Love et al. (2009), who showed a breakdown in automatic linking with slow speech. In fact, when looking at response times for the regular speech rate compared to the slow speech rate overall, results showed that in general, children with DLD were faster to respond in the slow rate condition than the regular rate condition. Though the important comparison of interest for this study is response times between the pronoun and control conditions within each rate of speech condition, the overall pattern further illustrates how slowed speech may facilitate real-time sentence processing in this group. Taken together, results suggest that children with DLD do in fact have knowledge of binding principles (since slowing down the sentence facilitated priming) and instead exhibit a language processing deficit in the speed at which they build these syntactic relationships.

We note here that children with DLD showed a similar pattern to TD children for processing of sentences containing reflexive anaphors at the regular rate of speech; that is, they did not show evidence of positive priming (which would indicate an incorrect link) upon encountering the reflexive anaphor. However, they differed from what has been published previously about priming patterns in TD children at the slow rate of speech. In Love et al. (2009), the authors found a marginally significant effect for priming of reflexives compared to control sentences and a significant effect when compared to pronouns, as TD children were quicker overall in responding in the reflexive condition (Love et al., 2009). The authors suggested that for TD children, the slow rate of speech caused breakdowns in the real-time linking that they demonstrated in the regular speech rate. However, for this study, the slow rate of speech facilitated children with DLD in making the *correct* pronoun-antecedent link while also causing them to *incorrectly* link the reflexive and the antecedent for the pronoun, indicating that slowed speech input aids performance for linguistic processes that are not yet acquired and impairs those that are already established. This dissociation in the benefit of slow speech suggests that children with DLD have knowledge of binding principles but follow a different developmental trajectory than TD children. It may be the case that it takes longer for children with DLD to develop the skills needed to acquire certain language processes than typically developing children, which implies that younger children with DLD might benefit more from slowed speech than older children with DLD. Future investigations are needed to test this possibility.

Thus far, the CMPP results indicate difficulties for children with DLD in automatic processing of sentences containing pronominal anaphors. This approach, however, cannot indicate if the slow rate of speech ultimately helps children with DLD with final comprehension of these sentence constructions. We now turn to Experiment (2) which explores overt, interpretative comprehension of sentences containing pronouns.

## 3 Experiment 2: sentence-picture matching (final sentence comprehension)

Experiment (2) used a sentence-picture matching task to investigate final comprehension of sentences requiring coreferential binding in children with DLD at both regular and slow rates of speech input. As previously discussed, prior research from Love et al. (2009) found that for final sentence comprehension, TD children demonstrated at-chance comprehension accuracy at the regular speech rate for the pronoun condition, but showed significant improvement when the rate of speech was slowed. Given that children with DLD did not show evidence of intact pronoun-antecedent linking at regular rates of speech, we hypothesize that they will perform at-chance with the interpretative task at regular rates of speech (with small gains with the slow rate of speech input). This hypothesis is based on the findings from Love et al. (2009) that conscious, final comprehension abilities are generally acquired after real-time pronoun linking abilities are acquired. In Experiment (1), children with DLD only showed evidence of facilitative priming in the pronoun condition once the rate of speech was slowed, indicating that they were unable to make co-referential links at regular rates of speech input during real-time sentence processing, which suggests they have not fully acquired the skill.

### 3.1 Materials and methods

#### 3.1.1 Participants

The same group of participants tested on the cross-modal picture priming task from Experiment (1) returned for 1 visit and were tested on the sentence-picture matching task. They remained in the same regular (*n* = 8) and slow (*n* = 6) rate groups for Experiment (2) (see Table 2).

#### 3.1.2 Study design and stimuli

**FIGURE 6:**
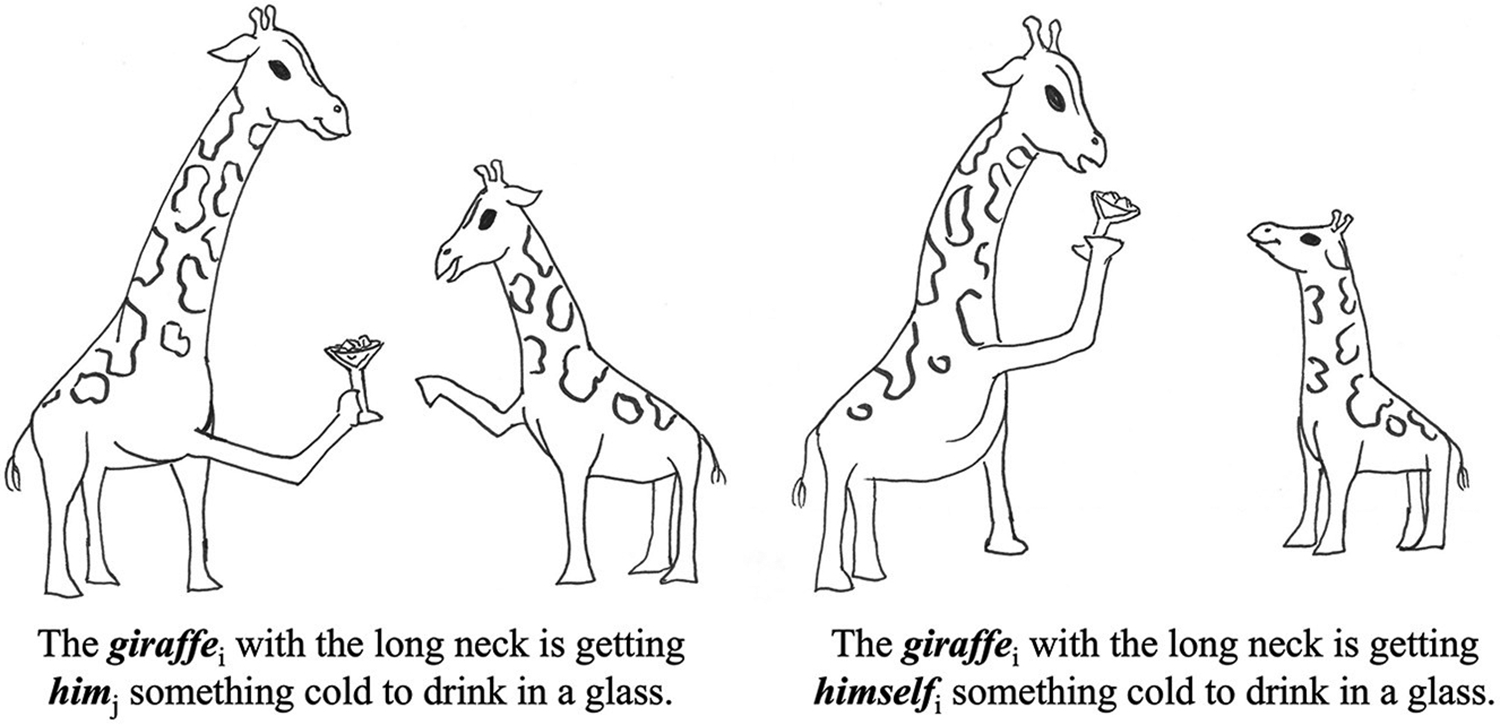
An example sentence and corresponding pictures for the sentence/picture matching task.

The design, materials, and procedure are the same as those used in Love et al. (2009) and are described in more detail therein. For the sake of brevity, we discuss the most relevant aspects here. Fifteen sentences from the Experiment (1) were selected for Experiment (2), based upon their high degree of imageability. Sentences were modified from Experiment (1) to only contain one noun phrase so that it limited participants’ ability to utilize pictorial context to interpret the referential links in the sentence. For each sentence, there were two versions, one with a pronoun and one with a reflexive, for a total of 30 items. Two images were created for each sentence; one correctly depicting a pronoun link and one correctly depicting a reflexive link (see Figure 6).

Experimental sentences were presented in a single session with the two versions presented at maximal distance. Sentences were recorded by a female native English speaker. In the regular rate of speech input condition, sentences were delivered at an average rate of 5.00 syllables per second. In the slow rate of speech condition, sentences were slowed to an average of 3.19 syllables per second (maintaining pitch, using the Cool Edit Pro v1.2 software package; Syntrillium Software, Phoenix, AZ). Each sentence was paired with the two pictures on an 8.5 × 11-inch picture card, the position of the pictures was counterbalanced across items. Each picture corresponded with either the pronoun or reflexive interpretation of the sentence.

#### 3.1.3 Procedure

Participants were instructed to listen carefully to the sentences they would hear and to select the picture that matched the sentence by pointing to it. During testing, sentences were presented to the participant via recorded audio files with the experimenter turning the picture cards before each recorded sentence was played. Children were familiarized with the task with a practice session consisting of four practice sentences before beginning the actual experiment.

#### 3.1.4 Analysis

Data were analyzed using a mixed-effects logistic regression model for binary accuracy data (correct or incorrect) with rate (regular, slow) and condition (pronoun, reflexive) as fixed effects and participant and sentence as random effects. Age was included as a covariate in the model. The model was estimated with SAS 9.4 (SAS Institute Inc, 2014) PROC MIXED. *Post-hoc* testing using least mean squares was used to examine significant main effects and the interaction for the model. *F*-statistics are reported for main effects and interactions and *t*-statistics are reported for follow-up comparisons.

### 3.2 Results

**FIGURE 7:**
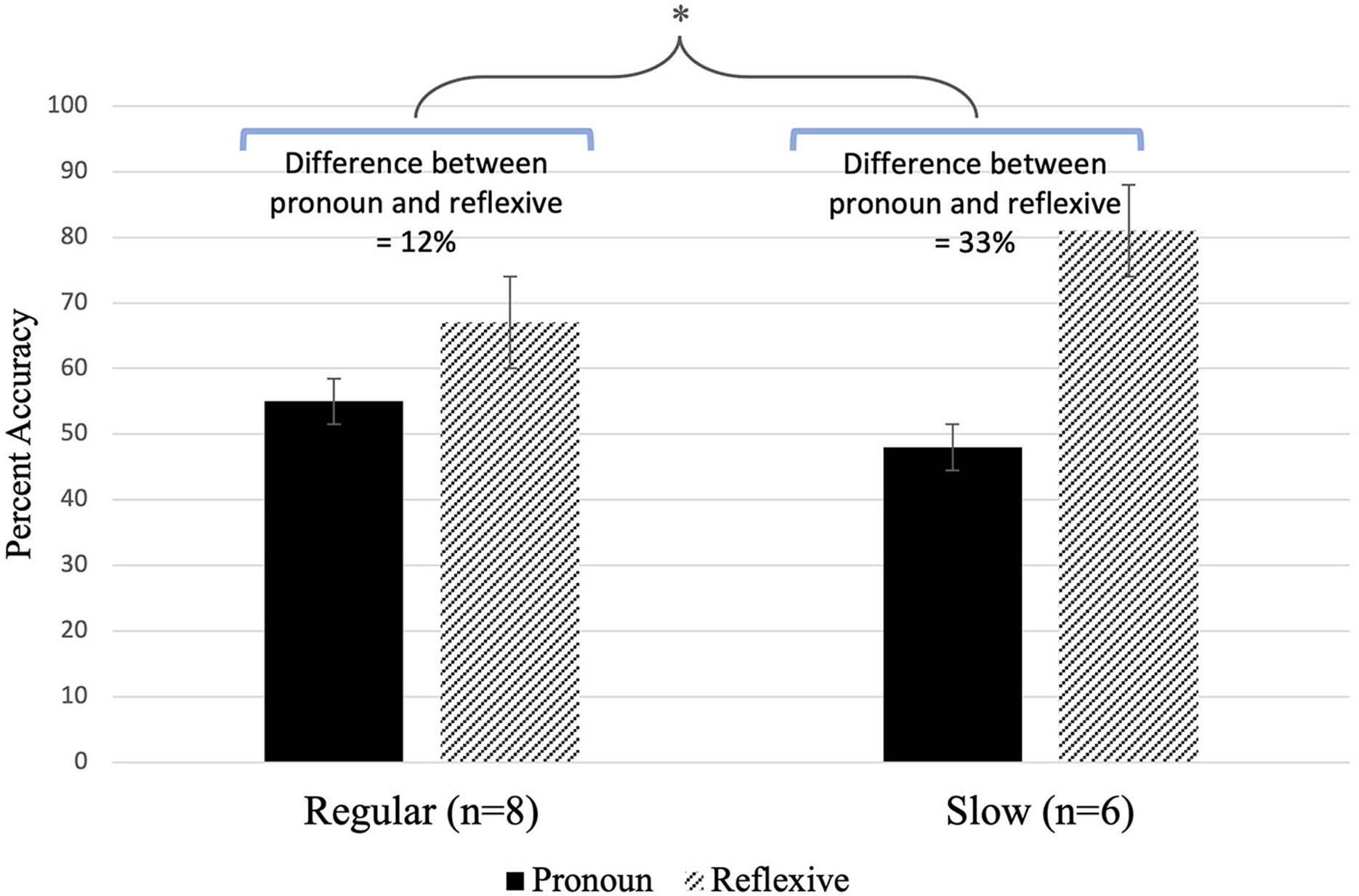
Percent accuracy (error bars are for standard error) for each rate condition. *indicates the significant rate x condition interaction effect for the difference in accuracy between the pronoun and reflexive conditions (blue brackets) when comparing the regular speech rate to the slow speech rate (*p* = 0.02).

For the comprehension accuracy data (Figure 7), a mixed-effects logistic regression model indicated that children with DLD did not demonstrate an overall *rate effect* between the regular (${\bar{x}}_{\text{accuracy}}=61\%$) and slow (${\bar{x}}_{\text{accuracy}}=64\%$) rates of speech [*F*_(1,415)_ = 0.01, *p* = 0.96]. However, consistent with prior research, the results revealed a significant main effect of *condition* [*F*_(1,415)_ = 23.80, *p* < 0.0001], indicating that across rates of speech, children with DLD were more accurate overall with sentences containing reflexives (${\bar{x}}_{\text{accuracy}}=73\%$) than sentences containing pronouns (${\bar{x}}_{\text{accuracy}}=52\%$); in fact, according to a one-sample *t*-test, performance in the pronoun condition was no different from chance [50%; *t*_(13)_ = 0.23, *p* = 0.82]. Importantly, further analysis revealed a significant *rate* × *condition* interaction [*F*_(1,415)_ = 5.52, *p* = 0.02]. This significant interaction arises from a large improvement in accuracy in the slow condition between pronoun (${\bar{x}}_{\text{accuracy}}=48\%$) and reflexive (${\bar{x}}_{\text{accuracy}}=81\%$) sentences [*t*_(415)_ = −4.78, *p* < 0.0001]; we note here that again, according to one-sample *t*-tests, performance for the pronoun condition was no different from chance for the regular [*t*_(7)_ = 0.52, *p* = 0.62] or slow [*t*_(5)_ = −0.16, *p* = 0.88] rate conditions. Overall, the slowed speech input helped improve comprehension accuracy for reflexives between the regular (${\bar{x}}_{\text{accuracy}}=67\%$) and slow (${\bar{x}}_{\text{accuracy}}=81\%$) conditions, but this difference did not reach statistical significance [$t_{\left(415\right)}=-1.60$, *p* = 0.11]. Additionally, as was the case in Experiment 1, there was a significant main effect of *age* in the model [*F*_(1,415)_ = 8.28, *p* = 0.004] for Experiment 2 suggesting that the older children were more accurate. However, again, as age was not the main focus of this study, more work is needed to carefully examine the effect of age and rate on final comprehension of sentences containing pronouns in children with DLD.

Of interest to this study was the difference in accuracy in sentences containing pronouns between the regular and slow rate speech conditions. While children with DLD performed better in understanding sentences containing pronouns in the regular rate condition (${\bar{x}}_{\text{accuracy}}=55\%$) as compared to the slow rate condition (${\bar{x}}_{\text{accuracy}}=48\%$), the difference was not significant [*t*_(415)_ = 1.67 *p* = 0.10]. We remind the reader that accuracy was not significantly different from chance (50%) for either condition. This pattern is partially consistent with our predictions, as we anticipated that children with DLD would demonstrate difficulty with the sentence-picture matching task regardless of speech rate. However, we also predicted that they would demonstrate a slight improvement in pronoun processing with the slowed speech rate. Instead, we found a (non-significant) decline in pronoun accuracy at the slow rate. Results indicate that slowing down the rate of speech did not help with conscious access of the linguistic knowledge required for successful pronoun-antecedent interpretation, despite evidence of real-time knowledge (i.e., priming with slow speech in Experiment 1) of binding principles.

### 3.3 Discussion: experiment 2

For experiment 2, we used a sentence-picture matching task to investigate final comprehension of sentences with either pronouns or reflexives. Here, we predicted that children with DLD would show impaired (at-chance) performance for identifying the correct picture in the pronoun condition, regardless of speech rate, but would see modest gains in accuracy at a slowed rate of speech. Partially consistent with this prediction, we found that for the pronoun condition, children performed at-chance at both regular and slow rates of speech but did not show any significant gains in accuracy for the slow rate.

This pattern differs from the results found for TD children in Love et al. (2009). In their study, TD children demonstrated at-chance performance for the interpretation of pronouns at a regular rate of speech but improved when speech was slowed, suggesting that the slower speech rate facilitated final comprehension of sentences containing pronouns. The authors concluded that slowed speech may help with final comprehension of sentences containing pronouns only when the metalinguistic processes necessary to link pronouns and antecedents have not yet been fully mastered, suggesting that slow speech may be more beneficial to younger children whose language skills are still developing.

The focus of this paper was on pronoun-antecedent linking, but it is important to note differences for reflexive-antecedent processing since it can provide insight into whether children with DLD have knowledge of different binding principles. In the sentence-picture-matching task, while children performed above chance in the reflexive condition, they were more accurate for the slow rate of speech compared to the regular rate of speech. The lack of ceiling effects in the data indicate that children with DLD have not yet fully mastered this ability, so when provided with more time, they perform better. The results from this study suggest that children with DLD have intact knowledge of binding (Principle A) but may need more time to reflect on and make the link between the reflexive and antecedent.

## 4 Limitations of the current study

The goal of this study was to examine real-time sentence processing in children with DLD. Here, we acknowledge the small sample size for each of the rates of speech. As is the case for most studies with clinical populations that are defined by strict inclusion guidelines, researchers should always aim to achieve sufficient power to identify effects that exist within the population, while still moving the field forward. Despite the small sample size, we believe this study provided an important initial exploration into the effects of rate of speech on sentence processing with children with DLD that will form a foundation for future studies.

Also, while not the initial focus of the study, preliminary findings indicate a significant effect of age in our group. Prior studies have demonstrated that mastery of reflexives occurs before pronouns, while real-time dependency linking abilities are acquired before dependency-linking abilities for final comprehension (Baauw and Cuetos, 2003; Love et al., 2009; McKee et al., 1993). If children with DLD demonstrate delays in these typical patterns, that can be mitigated by rate of speech, that would have important implications for future studies investigating different treatment approaches. Therefore, future studies should seek to include larger sample sizes that are carefully balanced across age to test how these patterns change over time and if children with DLD ever demonstrate typical, adult-like patterns of reaction time.

## 5 Conclusion

In sum, our results demonstrate that children with DLD show evidence of facilitative priming (i.e., are faster) for real-time pronoun-antecedent linking when speech input is slowed but do not show facilitation (i.e., are slower or show negative priming) when sentences are presented at a regular rate of speech. This pattern of performance is the opposite of what was observed previously in TD children (Love et al., 2009). This suggests that children with DLD may have a delay in processing these co-referential relationships for pronouns that can be mitigated by slowing down the incoming auditory speech stream. However, when the same group of children were tested on these pronoun-antecedent relationships using a sentence-picture matching task, they showed impaired (at-chance) interpretation of pronouns regardless of speech rate. This result is also different from that of TD children (Love et al., 2009), who showed improved accuracy in their final comprehension with a slowed rate of speech. Developmentally, the ability to link pronouns to their antecedents in real-time during sentence processing is acquired before the ability to link them for final sentence comprehension (Love et al., 2009; McKee et al., 1993; Schwartz et al., 2016). This likely has to do with the subconscious vs. conscious properties involved in real-time processing vs. final comprehension.

As stated previously, real-time processing methods measure the moment-by-moment mapping of words with their relationship to other words in the sentence, whereas final comprehension methods measure understanding after all different levels of processing are complete. Thus, these latter measures of accuracy encompass both sentence processing and sentence comprehension. For children with DLD, slowing down speech may help with real-time mapping of pronoun-antecedent relationships, but it may not be enough to mitigate the additional demands of final comprehension in terms of integrating all of the linguistic information, holding that information in memory, and making a final decision about the sentence meaning. In typically developing children, prior research has demonstrated a maturational effect of real-time vs. interpretative pronoun-antecedent linking, such that the former abilities are acquired before the latter abilities, again possibly due to the additional cognitive demands imposed by final comprehension tasks (Love et al., 2009). In addition to within-subject control investigations, future studies would benefit from larger sample sizes of children with DLD across age ranges to explore whether priming effects emerge at a later stage of development. More studies are also needed to understand how children with and without DLD can go from correctly parsing an uninterrupted sentence in real-time (as in Experiment 1, slow rate) to a failure in general comprehension (as in Experiment 2).

Taken together, the results from this study provide evidence that children with DLD do develop underlying knowledge of binding principles, which suggests that deficits in sentence processing may not be specifically linked to linguistic knowledge but instead are the result of other cognitive processes that impact language, such as delayed processing speed. The sentence triplet design used in our study allowed us to isolate pronominal binding while controlling for other factors such as sentence length and memory, which suggests that processing speed delays may more strongly impact language operations, though more work is needed to disentangle domain general processing from language-specific processing.

## Figures and Tables

**TABLE 1:** A summary of findings from Love et al. (2009), which investigated whether typically developing (TD) children would show facilitative priming during pronoun-antecedent linking at regular and slow rates of speech (in gray). Next to the summary are the hypotheses for the current study investigating the same processes in children with developmental language disorder (DLD).

	Regular rate		Slow rate	
	TD (Love et al., 2009)	DLD (current study hypothesis)	TD (Love et al., 2009)	DLD (current study hypothesis)
Cross-modal picture priming (response times)	Evidence of facilitative priming in pronoun condition	Will not show facilitative priming in pronoun condition	No evidence of facilitative priming in pronoun condition	Will show facilitative priming in pronoun condition
Final comprehension (accuracy)	Impaired (at-chance) accuracy in pronoun condition	Impaired (at-chance) accuracy in pronoun condition	Improved (above chance) accuracy in pronoun condition	Impaired (at-chance) accuracy with modest gains in pronoun condition

**TABLE 2:** Participant demographics and assessment scores [Mean (*Standard Deviation*)].

	TD children	Children with DLD		
	Love et al. (2009)	Regular (*n* = 8)	Slow (*n* = 6)	*p*-value
Age	7.43 *(1.80)*	7.42 *(1.79)*	8.32 *(1.20)*	0.28
Sex	21 females, 22 males	Four females, Four males	Four females, two males	–
Handedness	ND	Seven right, one left	Four right, one left, one both	–
CELF-4 core language index	115.26 *(10.10)*	71.88 *(9.48)*	75.33 *(15.81)*	0.65
TONI-3	113.67 *(12.10)*	95.00 *(13.14)*	105.80 *(16.24)*	0.25

ND, no data; CELF-4, Clinical Evaluation of Language Fundamentals, Version 4 (Semel et al., 2003); TONI-3, Test of Non-Verbal Intelligence, Version 3 (Brown et al., 1997); The CELF and the TONI are reported as scaled scores. The gray shaded region includes participant demographics for TD children tested by Love et al. (2009).

**TABLE 3:** The proportion of trials replaced for each participant following the winsorizing procedure. Winsorizing is used to reduce the effects of extreme values on mean response time (RT) (Wu and Zuo, 2009).

Participant	Rate	Proportion of trials replaced
001	Regular	0.10
002	Regular	0.04
003	Slow	0.03
004	Regular	0.05
005	Regular	0.04
006	Slow	0.07
007	Regular	0.00
008	Regular	0.02
009	Slow	0.02
010	Slow	0.00
011	Regular	0.01
012	Regular	0.08
013	Slow	0.04
014	Slow	0.04

**TABLE 4:** Mean response times (RT) in milliseconds and standard deviations (SD) for each condition by rate.

	Pronoun RT mean (SD)	Reflexive RT mean (SD)	Control RT mean (SD)
Regular (*n* = 8)	1,043 (328.0)	1,056 (342.4)	1,033 (305.9)
Slow (*n* = 6)	879 (278.8)	849 (260.3)	885 (291.7)

## Data Availability

The raw data supporting the conclusions of this article will be made available by the authors, without undue reservation.

## Footnotes

1 The indices “I”, “j”, and “k” represent a link between the pronoun and antecedent. The asterisk indicates a violation of binding principles. Note that “k” refers to a pronoun that does not have an antecedent within the sentence.
